# Chronic Obstructive Pulmonary Disease as an Independent Predictor of Left Main Coronary Artery Disease

**DOI:** 10.3390/medsci14010131

**Published:** 2026-03-11

**Authors:** Beatrice Ragnoli, Carlotta Bertelegni, Leonardo Brugiatelli, Tarsi Giovanni, Fausto Chiazza, Mario Malerba

**Affiliations:** 1 Respiratory Unit, S. Andrea Hospital, 13100 Vercelli, Italy; beatrice.ragnoli@uniupo.it; 2 Department of Translational Medicine, University of Piemonte Orientale, 28100 Novara, Italy; 3 Department of Pharmaceutical Sciences, University of Piemonte Orientale, 28100 Novara, Italy; 20037594@studenti.uniupo.it (C.B.); fausto.chiazza@uniupo.it (F.C.); 4 UOC Cardiologia—UTIC Ospedale S. Salvatore, AST 1, 61121 Pesaro, Italy

**Keywords:** chronic obstructive pulmonary disease, coronary artery disease, left main coronary artery, cardiovascular comorbidity, atherosclerosis, systemic inflammation

## Abstract

**Background:** Chronic obstructive pulmonary disease (COPD) is increasingly recognized as a disorder linked to increased cardiovascular risk, often coexisting with coronary artery disease (CAD), yet angiographic data on coronary involvement in COPD remain limited. This study aimed to evaluate whether COPD is associated with a distinct angiographic pattern of CAD, focusing on vessel distribution. **Methods:** We retrospectively enrolled 94 patients who underwent coronary angiography between 2023 and 2024 for suspected or known CAD. Clinical data, comorbidities, laboratory testing, pulmonary function, electrocardiography, echocardiography, and angiography were collected. Participants were stratified into two groups: COPD (*n* = 47) and non-COPD (*n* = 47). Coronary vessels were classified by number, location, and diameter. The normality of continuous variables was assessed using the Shapiro–Wilk test. Non-normally distributed variables were compared using the Mann–Whitney U test, while Fisher’s exact test was used for categorical comparisons. A multivariable logistic regression model was performed to identify independent predictors of left main coronary artery (LMCA) disease at the patient level. The primary endpoint was the association between COPD and CAD severity. **Results:** Baseline characteristics, including age, sex, BMI, and smoking history, were comparable between groups. The overall extent of CAD, expressed as the number of diseased vessels, did not differ significantly (*p* = 0.1436). However, vessel-based analysis revealed a distinct pattern: COPD patients showed a significantly higher prevalence of left main coronary artery (LMCA) disease compared to non-COPD patients (14% vs. 4.7%, *p* < 0.001). At the patient level, LMCA disease was present in 15/47 (31.9%) COPD patients compared with 6/47 (12.8%) non-COPD patients (*p* = 0.046). Multivariable logistic regression confirmed that COPD was an independent predictor of LMCA disease (OR = 3.56, 95% CI: 1.12–11.29, *p* = 0.031) after adjustment for age, sex, smoking, diabetes, and chronic kidney disease. Intermediate-caliber vessels were most frequently affected in both groups, while small-caliber branches were less commonly involved in COPD patients. **Conclusions**: COPD is an independent predictor of LMCA disease despite a similar overall angiographic extent of CAD. These findings suggest a distinct, high-risk coronary phenotype in COPD and highlight the need for enhanced cardiovascular vigilance and integrated cardiopulmonary management in this population.

## 1. Introduction

As two of the world’s most widespread chronic non-communicable conditions, chronic obstructive pulmonary disease (COPD) and coronary artery disease (CAD) are leading contributors to global morbidity and mortality [1,2,3,4,5,6]. A robust link between these diseases has been confirmed by recent studies demonstrating that increased COPD severity and the frequency of exacerbations correspond to a markedly higher risk of major cardiovascular events and death [7,8,9,10]. This connection extends beyond shared risk factors like advanced age and cigarette smoking, pointing to common underlying biological pathways—most notably systemic inflammation and endothelial dysfunction [11,12,13,14].

The clinical presentation of COPD and CAD can overlap considerably, with symptoms such as dyspnea and chest discomfort creating diagnostic ambiguity that may impede the timely identification and treatment of CAD in individuals with pre-existing lung disease [15]. This challenge carries profound prognostic weight, as CAD is the most common and impactful comorbidity in the COPD population, driving a large share of hospitalizations and deaths [16]. Conversely, COPD has been identified as an independent risk factor for cardiovascular mortality, even after accounting for smoking and other conventional risk factors [17,18].

While coronary angiography remains the definitive method for diagnosing CAD by enabling direct visualization of arterial stenosis, few studies have used this technique to map the specific angiographic features of CAD in the context of COPD, and the existing data are inconsistent [19]. Some reports suggest a greater prevalence of significant, multi-vessel stenosis and more severe atherosclerosis in patients with both conditions compared to those with CAD alone [20,21]. In contrast, other research has indicated a negative correlation between COPD and angiographically verified CAD [22,23,24,25]. These conflicting results reveal a significant gap in knowledge and underscore the need for further investigation.

Given the substantial prognostic implications and the risk of under-diagnosis and delayed treatment, a clearer understanding of the relationship between COPD and angiographically confirmed CAD holds direct clinical relevance. This study was therefore designed to characterize the association between COPD and the presence, severity, and anatomical distribution of CAD, as assessed by coronary angiography, in a cohort of patients undergoing the procedure for known or suspected coronary disease. By providing a detailed account of coronary involvement in this high-risk population, our objective is to generate findings that can support improved diagnostic precision and more effective therapeutic strategies.

## 2. Materials and Methods

### 2.1. Study Population

**Figure 1:**
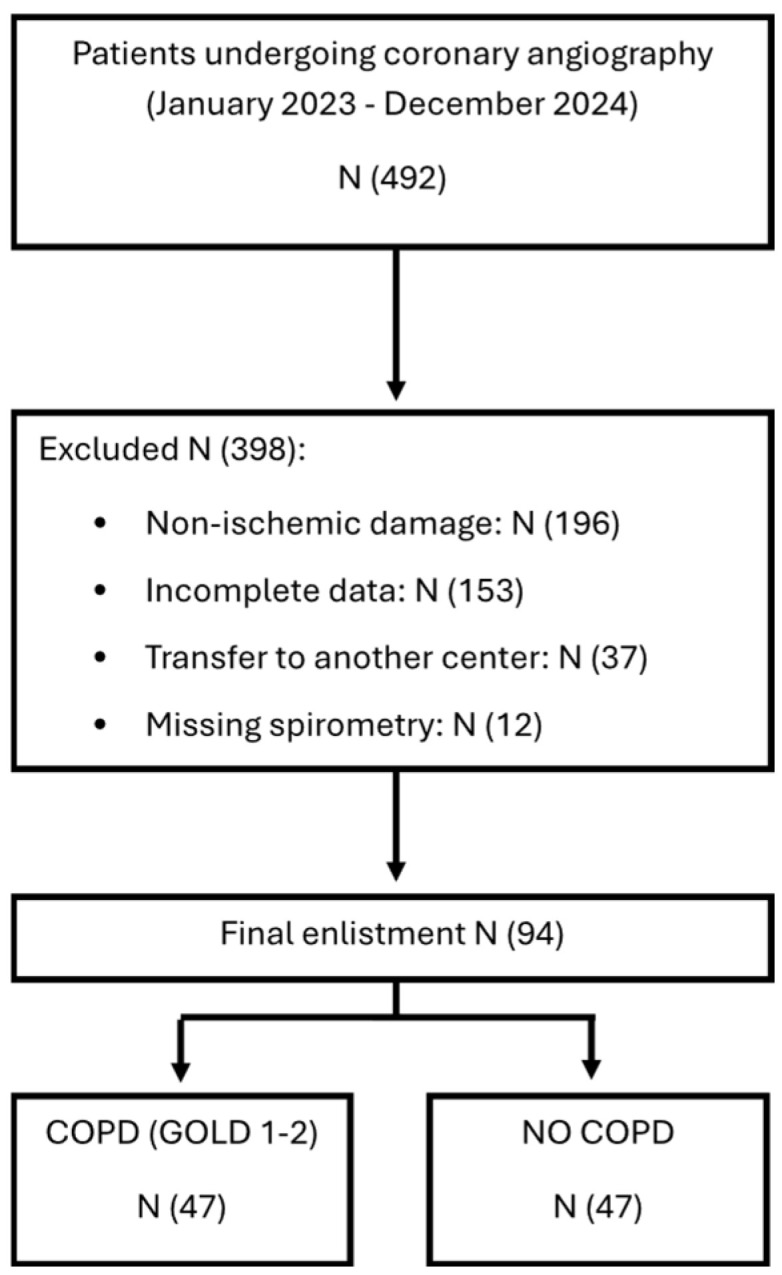
Flow diagram detailing patient screening, enrollment, and group allocation.

This retrospective observational single-center study enrolled 94 consecutive patients between January 2023 and December 2024 who underwent coronary angiography at San Salvatore Hospital, Pesaro, Italy. Patients were referred for invasive coronary evaluation because of suspected or known CAD, either in the setting of acute coronary syndrome (ACS) or chronic coronary syndrome (CCS). The Consolidated Standards of Reporting Trials (CONSORT) flow diagram detailing patient screening, enrollment, and group allocation is shown in Figure 1.

The diagnosis of COPD was established according to Global Initiative for Chronic Obstructive Lung Disease (GOLD) criteria based on post-bronchodilator spirometry [26]. Patients with GOLD stage 1–2 COPD were included, defined by a forced expiratory volume in 1 s (FEV_1_) > 80% or between 50% and 80% of the predicted value. The study was restricted to patients with GOLD stage 1–2 COPD to minimize the confounding effects of severe respiratory impairment and advanced systemic inflammation, which are more prevalent in GOLD 3–4 disease and could obscure the specific association between mild-to-moderate COPD and the initial stages of coronary atherosclerosis.

Exclusion criteria included a life expectancy < 6 months due to non-cardiovascular conditions and evidence of acute non-ischemic myocardial injury.

Obesity was defined as a body mass index (BMI) > 29 kg/m^2^. Hyperuricemia was defined as a serum uric acid concentration > 7 mg/dL. Chronic kidney disease was considered present in patients with stage 3b or higher (estimated glomerular filtration rate < 45 mL/min/1.73 m^2^).

All participants provided written informed consent prior to data collection and analysis. The study protocol was approved by the Institutional Ethics Committee (CE 67/20) and conducted in accordance with the principles of the Declaration of Helsinki.

### 2.2. Clinical Assessment and Diagnostic Workup

All patients underwent a thorough clinical evaluation that included the collection of demographic information, cardiovascular risk factors, comorbid conditions, and current pharmacological treatments. Documented therapies comprised dual antiplatelet regimens, prophylactic low-molecular-weight heparin (enoxaparin 4000 IU administered subcutaneously once daily), high-intensity statin therapy (rosuvastatin or atorvastatin), beta-adrenergic receptor antagonists (metoprolol or bisoprolol), and agents targeting the renin-angiotensin system (angiotensin-converting enzyme inhibitors or angiotensin receptor blockers).

A comprehensive laboratory panel was obtained for all patients, encompassing lipid parameters, inflammatory markers, and indices of renal function. Pulmonary function was assessed by spirometry to determine inspiratory capacity and FEV_1_. A standard 12-lead electrocardiogram was recorded using a Mortara ELI 280 device, and transthoracic echocardiography was performed with a Philips Epiq 7 system.

### 2.3. Clinical Presentation and Indication for Coronary Angiography

Patients presented either with ACS, including ST-segment elevation myocardial infarction (STEMI) and non-ST-segment elevation myocardial infarction (NSTEMI), or with CCS. In ACS without persistent ST-segment elevation, risk stratification was performed using the GRACE score, in accordance with European Society of Cardiology (ESC) guidelines [27].

Patients with unstable angina or NSTEMI were classified as very high risk or high risk based on ESC criteria. Very high-risk patients underwent immediate invasive evaluation (within 2 h), while high-risk patients underwent early invasive evaluation (preferably within 24 h). STEMI patients were referred directly to the catheterization laboratory for immediate reperfusion.

Patients with CCS underwent coronary angiography based on evidence of inducible ischemia documented by functional or imaging tests. Revascularization criteria were consistent with the ISCHEMIA trial and included left main coronary artery involvement, proximal left anterior descending artery disease, ischemic burden > 9%, or evidence of myocardial viability (≥11 segments on SPECT, ≥5 segments on stress echocardiography, or ≥3 segments on cardiac magnetic resonance imaging).

Some patients were referred for coronary angiography in the setting of decompensated congestive heart failure when ischemic etiology was suspected.

### 2.4. Coronary Angiography Assessment

Invasive coronary angiography was conducted following standard institutional protocols, which involved selective catheterization of the coronary arteries [28]. The resulting angiograms were reviewed to ascertain the presence, extent, and anatomical layout of CAD. For each patient, the total count of vessels with hemodynamically significant stenosis was documented. Coronary arteries were subsequently categorized based on their anatomical territory (left versus right circulation) and vessel caliber. As a vessel of particular prognostic importance, the left main coronary artery (diameter > 4 mm) was analyzed as a distinct category. Intermediate-caliber vessels (3–4 mm)—comprising the left anterior descending, left circumflex, and right coronary arteries—were grouped together, while all smaller-caliber branches (<3 mm) were classified as a separate category.

### 2.5. Endpoints and Statistical Analysis

The primary objective of this study was to evaluate the association between COPD and CAD, with specific focus on the number, anatomical distribution, and caliber of coronary vessels involved. The normality of continuous variables was assessed using the Shapiro–Wilk test. Variables with non-normal distribution were reported as median and interquartile range (IQR), and between-group comparisons were performed using the Mann–Whitney U test. Categorical variables were expressed as absolute number and percentage and compared using Fisher’s exact test.

To assess whether COPD was independently associated with left main coronary artery (LMCA) disease at the patient level, a multivariable logistic regression model was constructed. The dependent variable was the presence of LMCA disease (binary: yes/no), and the independent variables included COPD status, age, sex, smoking habit, diabetes mellitus, and chronic kidney disease. Results were expressed as odds ratios (ORs) with 95% confidence intervals (CIs). Model fit was assessed using the likelihood ratio test.

A *p*-value of <0.05 was considered statistically significant. All analyses were performed using JASP (version 0.19.3; JASP Team, University of Amsterdam, The Netherlands) and GraphPad Prism version 8.

## 3. Results

### 3.1. Demographic and Clinical Characteristics of the Study Population

A total of 94 patients were included in the study, of whom 47 had a confirmed diagnosis of COPD and 47 served as the non-COPD comparison group. The median age of the overall cohort was 78 years (IQR: 71–82), with no statistically significant difference between COPD and non-COPD patients (78 (74–84) vs. 77 (69–81) years, *p* = 0.1566). Most participants were male (71/94, 75.5%), and sex distribution was comparable between groups (74.5% vs. 76.6%, *p* > 0.9999).

Mean body mass index (BMI) was 23 kg/m^2^ (IQR: 21–28), with no significant differences between COPD and non-COPD (23 [22,23,24,25,26,27,28,29] vs. 23 [21,22,23,24,25,26,27] kg/m^2^, *p* = 0.6369). A history of current or former smoking was reported in 47 patients (50%), with similar prevalence in the COPD and non-COPD groups (53% vs. 47%).

Pulmonary function data were available for all patients, and showed a mean FEV_1_/FVC ratio of 68% (IQR: 63–70%) and a median FEV_1_ of 80% (IQR: 72–88%) predicted for COPD patients, consistent with GOLD stage 1–2 disease. The median BODE index in the COPD group was 4 (IQR: 3–4).

Laboratory parameters, including fibrinogen, lipid profile, C-reactive protein (CRP), and erythrocyte sedimentation rate (ESR), were comparable between groups. Cardiac functional assessment showed a mean left ventricular ejection fraction (LVEF) of 50% (IQR: 43–56%) in the overall cohort, with no significant difference between COPD and non-COPD patients. Estimated systolic pulmonary arterial pressure was also similar between groups (25 (20–35) vs. 25 (20–30) mmHg, *p* = 0.2804).

The clinical presentation leading to coronary angiography included STEMI, NSTEMI, and congestive heart failure (CHF), with distributions summarized in Table 1. Overall, baseline demographic, clinical, pulmonary, laboratory, and echocardiographic characteristics were well balanced between COPD and non-COPD patients.

Table 1 summarizes the demographic and clinical characteristics of the study population.

### 3.2. Cardiovascular Comorbidity Counts and Multimorbidity

**Figure 2:**
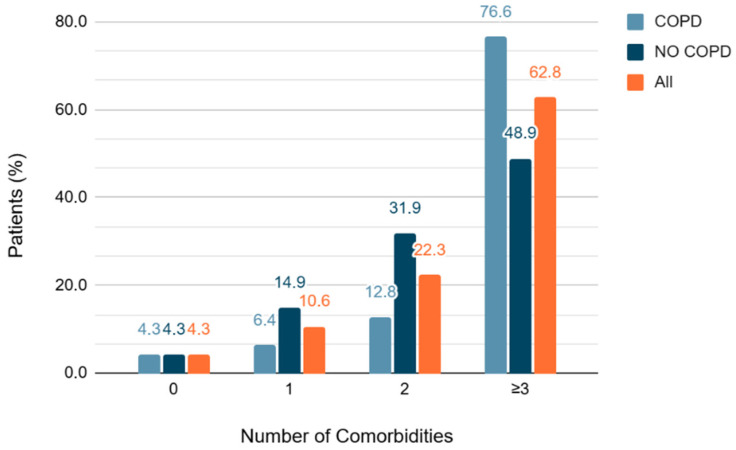
Number of cardiovascular comorbidities for patient in the two groups, with and without COPD and in all participants of the study. Abbreviation: COPD, chronic obstructive pulmonary disease).

The study population was characterized by a high burden of cardiovascular comorbidities. Only 4 individuals (4.3%) had no documented cardiovascular conditions, while the vast majority (90/94, 95.7%) had at least one. Notably, 59 patients (62.8%) carried three or more concurrent cardiovascular diagnoses. Arterial hypertension (79/94, 84%) and dyslipidemia (78/94, 83%) were the two most common conditions. Diabetes mellitus was documented in 31 patients (33%), obesity in 19 (20.2%), hyperuricemia in 24 (25.5%), and chronic kidney disease in 26 (28%). Additional cardiovascular conditions included peripheral arterial disease (12.8%), cerebrovascular disease (13.8%), and atrial fibrillation (29.8%). The distribution of these comorbidities was comparable between the COPD and non-COPD groups (Figure 2), with detailed data presented in Table 2.

### 3.3. Coronary Vessel Involvement

#### 3.3.1. Vessel-Based Angiographic Findings

Coronary angiography revealed differences in the distribution of vessel involvement between groups. Among COPD patients, two-vessel disease was most frequent (22/47, 47%), followed by three-vessel disease (14/47, 30%) and single-vessel disease (11/47, 23%). Conversely, non-COPD patients more commonly displayed three-vessel disease (21/47, 44.6%), with lower frequencies of two-vessel (13/47, 27.7%) and single-vessel disease (13/47, 27.7%). Overall, the distribution of the number of diseased vessels did not differ significantly between groups (*p* = 0.1436).

**Figure 3:**
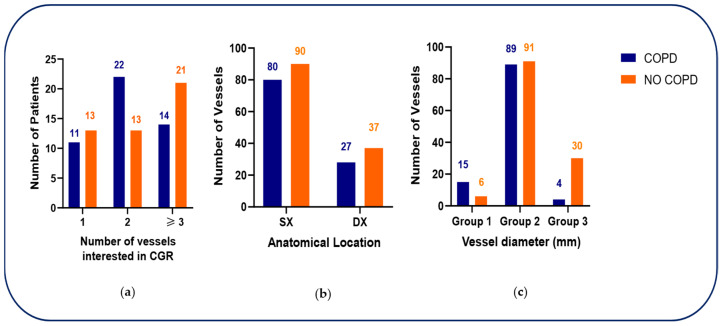
(**a**) Distribution of the number of diseased coronary vessels in patients with and without chronic obstructive pulmonary disease (COPD). (**b**) Distribution of coronary lesions according to anatomical circulation in patients with and without COPD (right vs. left coronary circulation). (**c**) Distribution of coronary lesions according to vessel diameter categories in patients with and without COPD. Abbreviations: COPD, chronic obstructive pulmonary disease; LAD, left anterior descending artery; LCX, left circumflex artery; RCA, right coronary artery; PDA, posterior descending artery. Vessel diameter categories: Group 1, left main coronary artery (diameter > 4 mm); Group 2, intermediate-caliber vessels (diameter 3–4 mm), including LAD, LCX, and RCA; Group 3, small-caliber branches (diameter < 3 mm), including PDA, diagonal branches, obtuse marginal branches, and posterolateral branches.

When coronary lesions were classified according to anatomical circulation, most lesions involved the left coronary circulation in both groups (74% in COPD vs. 71% in non-COPD), with no significant difference (*p* = 0.6612). In contrast, stratification by vessel diameter revealed a distinct pattern. In COPD patients, disease affecting the left main coronary artery (Group 1: LMCA; diameter > 4 mm) was significantly more frequent than in non-COPD patients (15/108 vessels, 14% vs. 6/127 vessels, 4.7%; *p* < 0.001). Intermediate-caliber vessels (Group 2: 3–4 mm), including the left anterior descending (LAD), left circumflex (LCX), and right coronary arteries (RCA), accounted for the majority of lesions in both groups (82% vs. 71.7%). Conversely, small-caliber branches (Group 3: < 3 mm) were less frequently involved in COPD patients compared with non-COPD patients (4% vs. 23.6%) (Figure 3).

Taken together, these findings indicate a disproportionate burden of left main CAD in patients with COPD, despite a similar overall extent of CAD as judged by the number of affected vessels. Detailed angiographic findings are reported in Table 3.

#### 3.3.2. Patient-Level Analysis and Predictors of LMCA Disease

To address the non-independence of vessel-level observations, a patient-level analysis of LMCA involvement was performed. LMCA disease was present in 15 out of 47 COPD patients (31.9%) compared with 6 out of 47 non-COPD patients (12.8%). This difference was statistically significant (Fisher’s exact test, *p* = 0.046; Table 4).

To determine whether COPD was independently associated with LMCA disease after adjustment for potential confounders, a multivariable logistic regression model was constructed. COPD status emerged as a significant independent predictor of LMCA disease (OR = 3.56, 95% CI: 1.12–11.29, *p* = 0.031), after adjustment for age, sex, smoking, diabetes mellitus, and chronic kidney disease. None of the other covariates reached statistical significance, although age showed a trend toward significance (OR = 1.07, 95% CI: 1.00–1.16, *p* = 0.060). Full regression results are presented in Table 5.

Taken together, these findings indicate that COPD is an independent predictor of LMCA disease, with patients with COPD having approximately 3.6-fold higher odds of LMCA involvement compared with non-COPD patients, even after controlling for major cardiovascular risk factors.

## 4. Discussion

In this study, we investigated the association between COPD and the severity of ischemic heart disease, assessed through coronary angiography. Although no significant differences were observed in the overall number of affected coronary vessels between patients with and without COPD, multivariable logistic regression analysis demonstrated that COPD is an independent predictor of left main coronary artery (LMCA) disease (OR = 3.56, 95% CI: 1.12–11.29, *p* = 0.031) after adjustment for age, sex, smoking, diabetes, and chronic kidney disease. This finding suggests that COPD may not be linked to a greater extent of coronary atherosclerosis per se, but rather to a distinct and prognostically unfavorable pattern of coronary involvement.

The association between COPD and ischemic heart disease is well established, with COPD recognized as an independent risk factor for cardiovascular events and cardiovascular mortality [8,23,24,29,30]. However, evidence derived from coronary angiographic studies has remained inconsistent [12]. While some studies have reported a higher prevalence of multivessel stenosis in COPD patients, others have failed to demonstrate meaningful differences in angiographic burden compared with non-COPD populations [19,23,24,25]. In this context, the present observation of a disproportionate involvement of LMCA in COPD patients adds a novel and clinically relevant element, given the well-known association between LMCA disease, adverse cardiovascular outcomes, and the need for timely and aggressive revascularization strategies.

Several pathophysiological mechanisms may underlie the preferential involvement of LMCA observed in COPD patients.

First, chronic systemic inflammation represents a central contributor. COPD is characterized by a persistent low-grade inflammatory state that extends beyond the pulmonary compartment and remains detectable even during clinical stability. This sustained inflammatory burden accelerates atherosclerotic progression and plaque vulnerability. Recent population-based studies have unveiled a strong association between systemic inflammatory markers in COPD and increased cardiovascular morbidity and mortality [8,31], supporting the hypothesis that inflammation-related vascular injury may preferentially affect large-caliber coronary vessels such as LMCA [32]. The independent association between COPD and LMCA disease observed in our multivariable analysis, after controlling for shared risk factors including smoking, diabetes, and chronic kidney disease, further supports the role of COPD-specific pathophysiological mechanisms—rather than traditional cardiovascular risk factors alone—in driving proximal coronary atherosclerosis.

Second, endothelial dysfunction and platelet activation represent additional mechanistic pathways linking COPD to coronary atherosclerosis. Chronic hypoxemia, oxidative stress, and systemic inflammation promote impaired nitric oxide bioavailability, endothelial injury, and enhanced platelet activation, amplifying vascular damage and atherothrombotic risk [33,34,35,36,37]. Furthermore, recent evidence indicates that endothelial dysfunction is particularly pronounced in COPD patients, especially in those with more severe airflow limitation or frequent exacerbations, further linking COPD pathophysiology to accelerated coronary atherosclerosis [9,38].

Third, hemodynamic alterations may also contribute to the preferential involvement of proximal coronary segments observed in patients with COPD. Lung hyperinflation and increased intrathoracic pressure, common in advanced disease, can impair diastolic coronary perfusion and modify cardiac geometry, thereby affecting coronary flow reserve [39,40,41,42,43]. These mechanical effects may disproportionately impact large-caliber proximal vessels such as the LMCA, which are particularly sensitive to changes in shear stress and transmural pressure. Although this mechanism has been less extensively investigated, analyses of cardiopulmonary and hemodynamic interactions support the hypothesis that COPD-related mechanical stress increases the vulnerability of proximal coronary segments [44,45].

In addition to these COPD-specific pathways, emerging biomarkers further reinforce this link. In particular, the monocyte-to-HDL ratio (MHR), recently proposed as a predictor of CAD specifically in COPD patients, reflects a pro-inflammatory and pro-atherogenic milieu that may predispose to more severe or proximally located coronary lesions [46]. Taken together, these observations support the hypothesis that COPD is associated with a distinct inflammatory–atherosclerotic phenotype, which may explain the higher prevalence of LMCA disease observed in our study.

The finding of more frequent LMCA involvement in COPD patients carries important clinical implications. Firstly, it underscores the need for earlier and more accurate cardiological evaluation in COPD patients, in whom symptoms of CAD may be masked by or misattributed to COPD, potentially leading to delayed diagnosis. Awareness of the increased likelihood of high-risk coronary lesions is therefore essential. Secondly, these data highlight the value of a multidisciplinary approach integrating pulmonologists and cardiologists to optimize diagnostic processes and therapeutic decision-making. Enhanced cardiovascular risk stratification may be particularly warranted in patients with more severe disease or frequent exacerbations, as LMCA involvement often requires revascularization, and COPD status may influence the choice between percutaneous coronary intervention and coronary artery bypass grafting due to increased perioperative risk. Finally, previous evidence indicating that COPD worsening is associated with a marked increase in cardiovascular risk [47] further reinforces the importance of close cardiac surveillance following exacerbation events.

This study has several strengths, including a detailed angiographic evaluation with vessel-specific and diameter-based classification, which allowed us to identify disease patterns not captured by conventional analyses. In addition, the careful matching of COPD and non-COPD groups reduces the impact of potential confounding factors. Furthermore, the patient-level multivariable logistic regression analysis strengthens the validity of our findings by demonstrating that the association between COPD and LMCA disease is independent of age, sex, smoking, diabetes, and chronic kidney disease, thereby addressing the potential limitation of confounding inherent in observational studies.

Nonetheless, several limitations should be acknowledged. The retrospective, single-center design inherently restricts the generalizability of our findings, and the relatively small sample size may have limited statistical power for selected comparisons. Moreover, the lack of stratification according to COPD severity or exacerbation history, and the inclusion of only GOLD 1–2 patients, precludes the evaluation of potential dose–response relationships and limits the generalizability of our findings to more severe COPD phenotypes. Finally, the absence of longitudinal follow-ups prevented us from determining the prognostic implications of the observed angiographic differences.

Future prospective, multicenter studies with larger cohorts, comprehensive clinical phenotyping, and long-term outcome data are thus needed to validate these results and to further elucidate the mechanisms linking COPD to specific patterns of CAD. In particular, future investigations incorporating molecular biomarkers of lung epithelial and endothelial cell injury, as well as markers of aberrant epithelial–mesenchymal crosstalk, will be essential to clarify the biological pathways through which COPD-related pulmonary damage may promote accelerated atherosclerosis in proximal coronary segments.

## 5. Conclusions

In conclusion, our study demonstrates that COPD is an independent predictor of left main coronary artery (LMCA) disease (OR = 3.56, 95% CI: 1.12–11.29). This finding suggests that COPD influences not just the risk of developing coronary atherosclerosis but its specific anatomical distribution, favoring the involvement of prognostically critical proximal segments. This observation holds true even after adjusting for major cardiovascular risk factors, pointing towards COPD-specific mechanisms, such as systemic inflammation and hemodynamic alterations, as key drivers of this high-risk coronary phenotype. Recognizing this distinct coronary phenotype is clinically important, as it may warrant more aggressive risk stratification and an integrated, multidisciplinary approach to management to mitigate the adverse outcomes associated with proximal coronary disease. Future studies incorporating longitudinal follow-up will be essential to determine whether this distinct coronary pattern translates into differential outcomes and to define optimal preventive and interventional strategies for this vulnerable population.

## Figures and Tables

**Table 1:** Demographic and clinical characteristics of the study population.

Variables	All (*n* = 94)	COPD (*n* = 47)	No COPD (*n* = 47)	*p*-Value
Age, years (median-IQR)	78 (71–82)	78 (74–84)	77 (69–81)	0.1566
Male sex, *n* (%)	71 (75.5)	35 (74.5)	36 (76.6)	>0.9999
BMI (Kg/m^2^)	23 (21–28)	23 (22–29)	23 (21–27)	0.6369
Smoking habitude, *n* (%)	57 (60.6)	32 (68.1)	25 (53.2)	0.2050
FEV1/FVC (% predicted)	71 (68–73)	68 (63–70)	73 (72–79)	<0.0001
FEV1 (% predicted)	85 (80–92)	80 (72–88)	88 (84–96)	<0.0001
BODE index	NA	4 (3–4)	NA	-
Fibrinogen (mg/dL)	482 (408–589)	481 (436–561)	483 (385–592)	0.5634
Cholesterol (mg/dL)	210 (198–228)	211 (200–226)	204 (198–229)	0.6244
LDL-C (mg/dL)	122 (105–140)	129 (112–141)	115 (99–140)	0.5611
HDL-C (mg/dL)	44 (40–55)	44 (40–56)	44 (40–55)	0.3153
Triglyceride (mg/dL)	135 (109–165)	140 (107–173)	133 (110–156)	0.5304
CRP (mg/dL)	0.44 (0.23–0.87)	0.44 (0.23–0.84)	0.39 (0.28–0.77)	0.2973
ESR	26 (17–39)	26 (17–40)	25 (17–39)	0.7141
EF %	50 (43–56)	50 (42–55)	55 (44–60)	0.3188
PAP systolic (mmHg)	25 (20–35)	25 (20–35)	25 (20–30)	0.2804
Presenting symptoms *n* (%)				
STEMI	11 (11.7)	5 (10.6)	6 (12.8)	0.8136
NSTEMI	44 (46.8)	21 (44.7)	23 (48.9)	
CCS	39 (41.5)	21 (44.7)	18 (38.3)	

Data are presented as medians and interquartile ranges (IQRs) for continuous variables. Abbreviations: *n* = (%) for categorical variables. BMI, body mass index; COPD, chronic obstructive pulmonary disease; CRP, c-reactive-protein; EF, ejection fraction; ESR, erythrocyte sedimentation rate; FEV1, forced expiratory volume in 1 s; FEV1/FVC, ratio of forced expiratory volume in 1 s to forced vital capacity; HDL-C, high-density lipoprotein cholesterol; LDL-C, low-density lipoprotein cholesterol; *n*, number of subjects; PAP, pulmonary arterial pressure; NA, not applicable. STEMI, ST-elevation myocardial infarction; NSTEMI, non-ST Elevation Myocardial Infarction; CCS, chronic coronary syndrome.

**Table 2:** Principal cardiovascular comorbidities of the study population.

Variable	All (*n* = 94)	COPD (*n* = 47)	No COPD (*n* = 47)	*p*-Value
Diabetes	31 (33)	11 (23)	20 (43)	0.0784
Arterial Hypertension	79 (84)	39 (83)	40(85)	0.9999
Dyslipidemia	78 (83)	38 (81)	40 (85)	0.7845
Obesity	19 (20.2)	11 (23.4)	8 (17)	0.6083
Hyperuricemia	24 (25.5)	10 (21)	14 (30)	0.4785
Kidney disease	26 (28)	14 (30)	12(26)	0.6489
AOP	12 (12.8)	6 (13)	6(13)	0.9999
Cerebrovascular disease	13 (13.8)	3 (6)	10 (21)	0.0700
FA	28 (29.8)	14 (29.8)	14 (29.8)	0.9999

Data are presented as *n* (%). Abbreviations: AOP, peripheral arterial disease; COPD, chronic obstructive pulmonary disease; FA, atrial fibrillation; *n*, number of subjects.

**Table 3:** Coronary angiographic characteristics according to COPD status.

Variable	COPD (*n* = 47)	no COPD (*n* = 47)	*p*-Value
Number of vessels			
1	11 (23)	13 (27.7)	
2	22 (47)	13 (27.7)	0.1436
≥3	14 (30)	21 (44.6)	
Variables	COPD (nv = 108)	no COPD (nv = 127)	*p*-value
Anatomical location			
Dx	28 (26)	37 (29)	0.6612
Sx	80 (74)	90 (71)	
Diameter of vessels			
Group 1	15 (14)	6 (4.7)	<0.0001
Group 2	89 (82)	91 (71.7)	
Group 3	4 (4)	30 (23.6)	

Data are presented as *n* (%). Abbreviations: COPD, chronic obstructive pulmonary disease; *n*, number of subjects; nv, number of vessels. Vessel diameter groups: Group 1, left main coronary artery (diameter > 4 mm); Group 2, intermediate-caliber vessels (3–4 mm), including LAD, LCX, and RCA; Group 3, small-caliber branches (diameter < 3 mm), including posterior descending artery, diagonal branches, obtuse marginal branches, and posterolateral branches.

**Table 4:** Patient-level analysis of left main coronary artery (LMCA) involvement according to COPD status.

	LMCA Absent	LMCA Present	Total	*p*-Value
COPD (*n* = 47)	32 (68.1%)	15 (31.9%)	47	
Non-COPD (*n* = 47)	41 (87.2%)	6 (12.8%)	47	0.046 *
Total	73	21	94	

Data are presented as *n* (%). * Fisher’s exact test. Abbreviations: COPD, chronic obstructive pulmonary disease; LMCA, left main coronary artery.

**Table 5:** Multivariable logistic regression analysis for predictors of left main coronary artery (LMCA) disease.

Variable	Estimate	Standard Error	Odds Ratio	z	*p*-Value	95% CI Lower	95% CI Upper
Intercept (M_0_)	−1.246	0.248	0.288	−5.032	<0.001	0.177	0.467
Intercept (M_1_)	−8.187	3.226	2.783 × 10^−4^	−2.538	0.011	0.000	0.155
Age	0.071	0.038	1.073	1.881	0.060	0.997	1.156
Sex (male)	0.348	0.633	1.417	0.550	0.582	0.410	4.894
Smoking	0.552	0.577	1.737	0.957	0.338	0.561	5.379
Diabetes	0.815	0.627	2.259	1.299	0.194	0.661	7.723
CKD	−0.655	0.653	0.520	−1.002	0.316	0.144	1.869
**COPD status**	**1.270**	**0.589**	**3.561**	**2.157**	**0.031**	**1.123**	**11.294**

Note. LMCA status level ‘1’ coded as class 1. Dependent variable: LMCA disease (present = 1, absent = 0). M_0_ = null model (intercept only); M_1_ = full model with covariates. Abbreviations: CI, confidence interval; CKD, chronic kidney disease; COPD, chronic obstructive pulmonary disease; LMCA, left main coronary artery.

## Data Availability

The raw data supporting the conclusions of this article will be made available by the authors on request.

## Abbreviations

The following abbreviations are used in this manuscript:

COPD	Chronic Obstructive Pulmonary Disease
CAD	Coronary Artery Disease
GOLD	Global Initiative for Chronic Obstructive Lung Disease
BMI	Body Mass Index
LMCA	Left Main Coronary Artery
ACS	Acute Coronary Syndrome
CCS	Chronic Coronary Syndrome
FEV1	Forced Expiratory Volume in 1 s
IU	International Unit
STEMI	ST-segment Elevation Myocardial Infarction
NSTEMI	non-ST-segment Elevation Myocardial Infarction
GRACE	Global Registry of Acute Coronary Events
ESC	European Society of Cardiology
SPECT	Single Photon Emission Computed Tomography
SD	Standard Deviation
FVC	Forced Vital Capacity
FEV1/FVC	Tiffeneau Index
BODE	Body-mass index, Obstruction, Dyspnea, Exercise
CRP	C-Reactive Protein
ESR	Erythrocyte Sedimentation Rate
LVEF	Left Ventricular Ejection Fraction
LAD	Left Anterior Descending
LCX	Left Circumflex
RCA	Right Coronary Artery
MHR	Monocyte-to-HDL Ratio

## References

[1] Wang Y., Han R., Ding X., Feng W., Gao R., Ma A. (2025). Chronic obstructive pulmonary disease across three decades: Trends, inequalities, and projections from the Global Burden of Disease Study 2021. Front. Med..

[2] de Oca M.M., Perez-Padilla R., Celli B., Aaron S.D., Wehrmeister C.F., Amaral A.F.S., Mannino D., Zheng J., Salvi S., Obaseki D. (2024). The global burden of COPD: Epidemiology and effect of prevention strategies. Lancet Respir. Med..

[3] Lindstrom M., DeCleene N., Dorsey H., Fuster V., Johnson C.O., LeGrand K.E., Mensah G.A., Razo C., Stark B., Turco J.V. (2023). Global Burden of Cardiovascular Diseases and Risks Collaboration, 1990–2022. J. Am. Coll. Cardiol. (JACC).

[4] Pant S. (2025). Cardiovascular conditions lead global disease burden over the past 30 years. JAMA.

[5] Boers E., Allen A., Barrett M., Benjafield A.V., Rice M.B., Wedzicha J.A., Kaye L., Zar H.J., Sinha S., Ozoh O. (2025). Forecasting the Global Economic and Health Burden of COPD from 2025 Through 2050. Chest.

[6] Stark B.A., DeCleene N.K., Desai E.C., Hsu J.M., Johnson C.O., Lara-Castor L., LeGrand K.E., A B., Aalipour M.A., Aalruz H. (2025). Global, Regional, and National Burden of Cardiovascular Diseases and Risk Factors in 204 Countries and Territories, 1990–2023. J. Am. Coll. Cardiol..

[7] Nowbar A.N., Gitto M., Howard J.P., Francis D.P., Al-Lamee R. (2019). Mortality from Ischemic Heart Disease Analysis of Data from the World Health Organization and Coronary Artery Disease Risk Factors from NCD Risk Factor Collaboration. Circ. Cardiovasc. Qual. Outcomes.

[8] Li Y., Li F., Wang G., Zeng Q., Xie P. (2025). Additive impact of chronic obstructive pulmonary disease (COPD) and cardiovascular disease (CVD) on all-cause and disease-Specific mortality: A longitudinal nationwide population-based study. BMC Pulm. Med..

[9] Yang H., Ryu M.H., Carey V.J., Young K., Kinney G.L., Dransfield M.T., Wade R.C., Wells J.M., Budoff M.J., Castaldi P.J. (2024). Differential Association of COPD Subtypes with Cardiovascular Events and COPD Exacerbations. Chest.

[10] Morgan C., Challen R., Begier E., Southern J., Nava G., Qian G., McGuinness S., King J., Maskell N., Lahuerta M. (2025). Acute coronary syndrome after an infective exacerbation of COPD: A prospective cohort study of acute lower respiratory tract disease in hospitalised adults. ERJ Open Res..

[11] Ragnoli B., Chiazza F., Tarsi G., Malerba M. (2025). Biological pathways and mechanisms linking COPD and cardiovascular disease. Ther. Adv. Chronic Dis..

[12] Papaporfyriou A., Bartziokas K., Gompelmann D., Idzko M., Fouka E., Zaneli S., Bakakos P., Loukides S., Papaioannou A. (2023). Cardiovascular diseases in COPD: From Diagnosis and Prevalence to Therapy. Life.

[13] Jesus F.R., Passos F.C., Lopes Falcão M.M., Sarno Filho M.V., Neves da Silva I.L., Moraes A.C.S., Neves M.C.L.C., Baccan G.C. (2024). Immunosenescence and Inflammation in Chronic Obstructive Pulmonary Disease: A Systematic Review. J. Clin. Med..

[14] Marcuccio G., Candia C., Maniscalco M., Ambrosino P. (2025). Endothelial dysfunction in chronic obstructive pulmonary disease: An update on mechanisms, assessment tools and treatment strategies. Front. Med..

[15] Beyer C., Pizzini A., Boehm A., Loeffler-Ragg J., Weiss G., Feuchtner G., Bauer A., Friedrich G., Plank F. (2020). Underappreciation of coronary artery disease in patients with COPD. Eur. Respir. J..

[16] Cao Z., He L., Luo Y., Tong X., Zhao J., Huang K., Chen Q., Jiao L., Liu Y., Geldsetzer P. (2025). Burden of chronic respiratory diseases and their attributable risk factors in 204 countries and territories, 1990–2021: Results from the global burden of disease study. Chin. Med. J. Pulm. Crit. Care Med..

[17] Lin W., Chen C., Lai C.H. (2017). Long-term outcomes of percutaneous coronary intervention in patients with chronic obstructive pulmonary disease: A 13-year nationwide cohort study. Eur. Respir. J..

[18] Zheng Y., Hu Z., Seery S., Li C., Yang J., Wang W., Qi Y., Shao C., Fu Y., Xiao H. (2024). Global Insights into Chronic Obstructive Pulmonary Disease and Coronary Artery Disease: A Systematic Review and Meta-Analysis of 6,400,000 Patients. Rev. Cardiovasc. Med..

[19] Hong Y., Graham M.M., Southern D., McMurtry M.S. (2019). The Association between Chronic Obstructive Pulmonary Disease and Coronary Artery Disease in Patients Undergoing Coronary Angiography. J. Chronic Obstr. Pulm. Dis..

[20] Pereira Ferreira E.J., de Carvalho Cardoso L.V., de Matos C.J.O., Mota I.L., Lira J.M.C., Gomes Lopes M.E., Santos G.V., Almeida M.L.D., Aguiar-Oliveira M.H., Sobral Sousa A.C.S. (2023). Cardiovascular Prognosis of Subclinical Chronic Obstructive Pulmonary Disease in Patients with Suspected or Confirmed Coronary Artery Disease. Int. J. Chronic Obstruct. Pulm. Dis..

[21] Williams M.C., Murchison J.T., Edwards L.D., Agustí A., Bakke P., Calverley P.M.A., Celli B., Coxson H.O., Crim C., Lomas D.A. (2014). Coronary artery calcification is increased in patients with COPD and associated with increased morbidity and mortality. Thorax.

[22] Pavasini R., Campo G. (2025). Complex coexistence of COPD and cardiovascular disease. Thorax.

[23] Polmana R., Hurst J.R., Faruk Uysal O., Mandal S., Linz D., Simons S. (2024). Cardiovascular disease and risk in COPD: A state of the art review. Expert. Rev. Cardiovasc. Ther..

[24] Sá-Sousa A., Rodeigues C., Jacome C., Cardoso J., Fortuna I., Guimarães M., Pinto P., Sarmento P.M., Baptista R. (2024). Cardiovascular Risk in Patients with COPD: A Systematic Review. J. Clin. Med..

[25] Bellou V. (2025). Quantitative Imaging to Illuminate Cardiovascular Risk in COPD—Progress, Context, and the Path Ahead. Int. J. Chronic Obstruct. Pulm. Dis..

[26] Kostikas K., Hillas G., Gogali A. (2025). 2025 GOLD Report: What is New and What is Noteworthy for the Practicing Clinician. COPD J. Chronic Obstr. Pulm. Dis..

[27] Byrne R.A., Rossello X., Coughlan J.J., Barbato E., Berry C., Chieffo A., Claeys M.J., Dan G.A., Dweck M.R., Galbraith M. (2023). 2023 ESC Guidelines for the management of acute coronary syndromes. Eur. Heart J..

[28] Rao S.V., O’Donoghue M.L., Ruel M., Rab T., Tamis-Holland J.E., Alexander J.H., Baber U., Baker H., Cohen M.G., Cruz-Ruiz M. (2025). 2025 ACC/AHA/ACEP/NAEMSP/SCAI Guideline for the Management of Patients with Acute Coronary Syndromes: A Report of the American College of Cardiology/American Heart Association Joint Committee on Clinical Practice Guidelines. Circulation.

[29] Nordon C., Simons S.O., Marshall J., Müllerová H., Pollack M., Bengtsson C., Hoti F., Lobier M., Salosensaari A., Santos A.C. (2025). The sustained increase of cardiovascular risk following COPD exacerbations: Meta-analyses of the EXACOS-CV studies. ERJ Open Res..

[30] Gale C.P., Hurst J.R., Hawkins N.M., Bourbeau J., Han M.K., Lam C.S.P., Marciniuk D.D., Price D., Stolz D., Gluckman T. (2025). Identification and management of cardiopulmonary risk in COPD: A multidisciplinary consensus. Eur. J. Prev. Cardiol..

[31] Li T., Chen L., Xu H., Zheng Y., Yang H., Zhao H., Chen C. (2025). The association between cardiovascular diseases and their subcategories with the severity of COPD: A large cross-sectional study. Front. Cardiovasc. Med..

[32] Soehnlein O., Lutgens E., Döring Y. (2025). Distinct inflammatory pathways shape atherosclerosis in different vascular beds. Eur. Heart J..

[33] Malerba M., Nardin M., Radaeli A., Montuschi P., Carpagnano G.E., Clini E. (2017). The potential role of endothelial dysfunction and platelet activation in the development of thrombotic risk in COPD patients. Expert. Rev. Respir. Med..

[34] Siragusa S., Natali G., Nogara A.M., Trevisani M., Lagrasta C.A.M., Pontis S. (2023). The role of pulmonary vascular endothelium in chronic obstructive pulmonary disease (COPD): Does endothelium play a role in the onset and progression of COPD?. Explor. Med..

[35] Marques P., Bocigas I., Domingo E., Francisco V., Tarraso J., Garcia-Sanjuan Y., Morcillo E.J., Piqueras L., Signes-Costa J., Gonzales C. (2024). Key role of activated platelets in the enhanced adhesion of circulating leucocyte–platelet aggregates to the dysfunctional endothelium in early-stage COPD. Front. Immunol..

[36] Kazemi N., Bordbar A., Bavarsad S.S., Ghasemi P., Bakhshi M., Rezaeeyan H. (2024). Molecular insights into the relationship between platelet activation and endothelial dysfunction: Molecular approaches and clinical practice. Mol. Biotechnol..

[37] Pistenmaa C.L., Hoffman E.A., Prince M.R., Hughes E., Dashnaw S., Lo Cascio C.M., Oelsner E.C., Shen W., Sun Y., Winther H. (2025). Platelet activation and COPD-related clinical and imaging characteristics: The Multi-Ethnic Study of Atherosclerosis (MESA) COPD Study. Respir. Med..

[38] MacLeod M., Knott K., Lopez R., Bartlett-Pestell S., Braddy-Green A., Khamis R.Y., Barnes P.J., Collins P., Nicol E.D., Wedzicha J.A. (2024). Endothelial dysfunction and coronary artery disease in patients with COPD. Eur. Respir. J..

[39] Wells J.M., Washko G.R., Han M.K., Abbas N., Nath H., Mamary A.J., Regan E., Bailey W.C., Martinez F.J., Westfall E. (2012). Pulmonary arterial enlargement and acute exacerbations of COPD. N. Engl. J. Med..

[40] Koopman M., Posthuma R., Vanfleteren L.E.G.W., Simons S.O., Franssen F.M.E. (2024). Lung Hyperinflation as Treatable Trait in Chronic Obstructive Pulmonary Disease: A Narrative Review. Int. J. Chronic Obstruct. Pulm. Dis..

[41] Xu Y., Yamashiro T., Moriya H., Tsubakimoto M., Tsuchiya N., Yukihiro N., Matsuoka S., Murayama S. (2017). Hyperinflated lungs compress the heart during expiration in COPD patients: A dynamic-ventilation CT study. Int. J. Chronic Obstruct. Pulm. Dis..

[42] Watz H., Waschki B., Meyer T., Kretschmar G., Kirsten A., Claussen M., Magnussen H. (2018). Decreased cardiac chamber sizes and associated clinical outcomes in COPD. JACC Cardiovasc. Imaging.

[43] Kohli P. (2016). Lung Hyperinflation and Cardiac Impairment in Chronic Obstructive Pulmonary Disease. Master’s Thesis.

[44] MacLeod M.A., Knott K.D., Allinson J.P., Finney L.J., Wiseman D.J., Ritchie A.I., Braddy-Green A., Barlett-Pestell S., Lopez R., Sun L. (2024). Prevalence and Clinical Correlates of Radiologically Detected Coronary Artery Disease in Chronic Obstructive Pulmonary Disease: A Cross-Sectional Observational Study. Am. J. Respir. Crit. Care Med. AJRCCM.

[45] Soehnlein O., Libby P. (2021). Targeting inflammation in atherosclerosis—From experimental insights to the clinic. Nat. Rev. Drug Discov..

[46] Sun F., Ye M., Jumahan A., Aainiwaier A., Xia Y. (2025). MHR as a promising predictor for coronary artery disease in COPD patients: Insights from a retrospective nomogram study. Respir. Med..

[47] Pirera E., Di Raimondo D., D’Anna L., Tuttolomondo A. (2025). Risk trajectory of cardiovascular events after an exacerbation of chronic obstructive pulmonary disease: A systematic review and meta-analysis. Eur. J. Intern. Med..

